# A rare case of *Pseudomonas putida* ventriculitis in intensive care unit: A case report

**DOI:** 10.3389/fmed.2022.1058121

**Published:** 2022-12-08

**Authors:** Mohammad Nizam Mokhtar, Izzuddin Azaharuddin, Farah Hanim Abdullah, Azarinah Izaham, Raha Abdul Rahman

**Affiliations:** Department of Anaesthesiology and Intensive Care, Faculty of Medicine, Universiti Kebangsaan Malaysia, Kuala Lumpur, Malaysia

**Keywords:** *Pseudomonas putida*, ventriculitis, meningitis, nosocomial infection, external ventricular drain (EVD)

## Abstract

*Pseudomonas putida* is a rare pathogen leading to nosocomial and central nervous system infections. Despite having a low virulence and being a rare organism to cause bacteremia, it can evolve into a multidrug-resistant organism and lead to mortality and morbidity in the intensive care setting. A 64-year-old male gardener was presented with extensive acute subarachnoid hemorrhage with intraventricular extension causing hydrocephalus requiring embolization and coiling following a cerebral angiogram, which showed bilateral posterior circulation aneurysm and left anterior circulation aneurysm. External ventricular drain (EVD) was inserted given the worsening hydrocephalus. During his stay in the intensive care unit (ICU), he was becoming more septic and a full septic workup including a cerebral spinal fluid culture taken from the indwelling catheter of the EVD and was found to be positive for a ceftazidime-sensitive strain of *P. putida*. Following the treatment with intravenous ceftazidime for 1 week and a revision of the EVD on day 32 of admission, he continued to recover well and showed an improvement in his Glasgow Coma Scale (GCS) and septic parameters. Eventually, he was able to wean off mechanical ventilation. He was discharged from ICU care to the neurosurgical ward with supplemental oxygen on day 42 of admission. It is necessary to be aware of the possibility of nosocomial *P. putida* infection, especially in patients with indwelling catheters, and to consider the early initiation of appropriate antibiotic regimens once detected as well as strict precautions in hygiene during the management of these patients to avoid further development of multi-drug resistant (MDR) strains.

## Introduction

*Pseudomonas putida* is a member of the fluorescent group of pseudomonas (1). *P. putida* is a Gram-negative rod-shaped bacterium that is seen in soil, water, and moist environments. It may colonize the skin and may lead to opportunistic infections in immunocompromised patients. *P. putida* is a rare pathogen leading to nosocomial infections (2). Despite its low virulence, it can evolve into multi-drug resistant (MDR) organisms and lead to mortality and morbidity in the intensive care setting (2). In our local setting, we have encountered only three cases of *P. putida* infection in the past 5 years, two of which were bacteremia in immunocompromised patients, therefore, the prevalence of *P. putida* in our intensive care was found to be less than 0.1% of all Gram-negative infections. We report a case of ventriculitis with *P. putida* occurring in an immunocompetent host following a neurosurgical procedure.

## Case presentation

**FIGURE 1:**
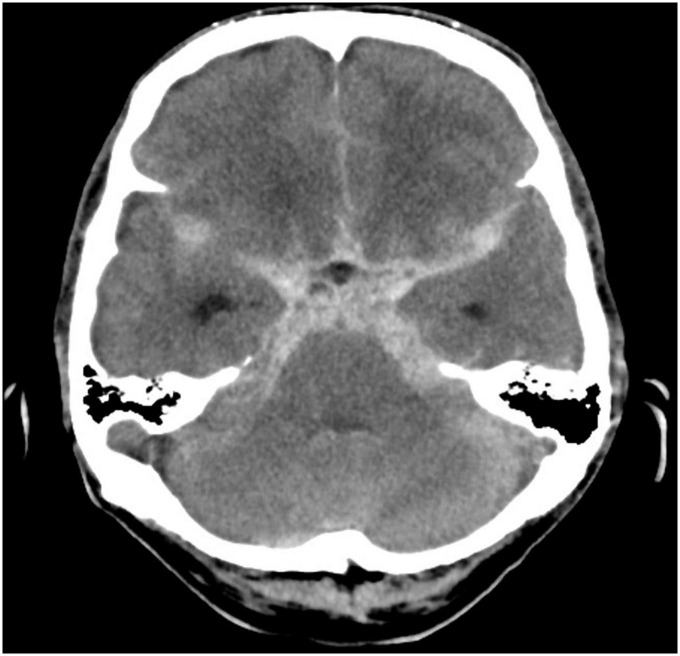
Computed tomography brain showing extensive acute subarachnoid hemorrhage.

**FIGURE 2:**
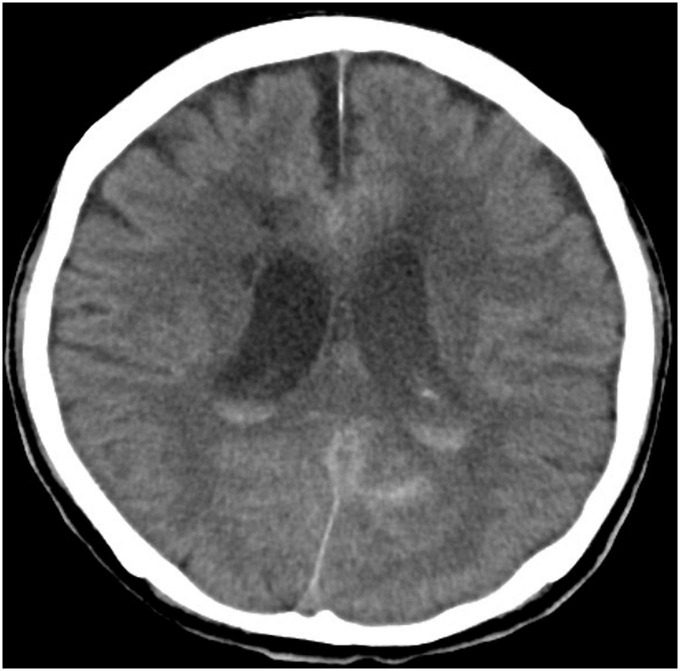
Computed tomography brain showing intraventricular extension leading to hydrocephalus.

**FIGURE 3:**
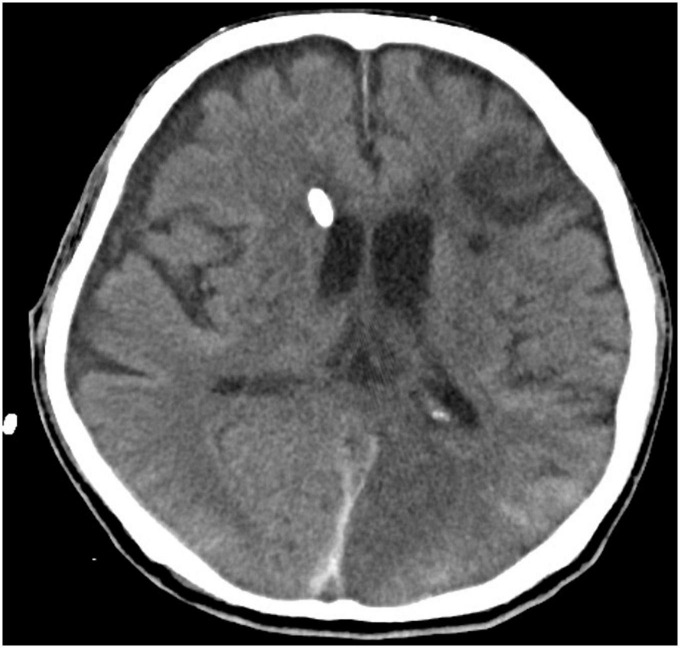
Computed tomography brain with EVD traversing through the right lateral ventricle with reduction of hydrocephalus.

A 64-year-old male gardener presented to the emergency department with hypertensive emergency and reduced consciousness. Computed Tomography (CT) brain upon admission revealed extensive acute subarachnoid hemorrhage with intraventricular extension causing hydrocephalus (Figures 1, 2). He was admitted into our intensive care unit (ICU) following an external ventricular drain (EVD) insertion procedure to treat the hydrocephalus (Figure 3). The following day, he underwent cerebral aneurysm embolization and coiling following a cerebral angiogram, which showed a bilateral posterior circulation aneurysm and left anterior circulation aneurysm. Upon ICU admission, cerebral resuscitation was initiated and multiple attempts of weaning and extubation throughout a period of 2 weeks were done. Despite that, he had poor Glasgow Coma Scale (GCS) recovery, following which a tracheostomy was performed on day 21 of admission.

Subsequently, he became more septic with worsening hemodynamics requiring intravenous noradrenaline infusion to achieve a mean arterial pressure (MAP) of 80 mmHg and the increasing ventilatory requirement to maintain adequate oxygenation. He was treated for ventilator-associated pneumonia (VAP) evidenced by *Acinetobacter* sp. isolated from the tracheal aspirates. Despite the resolution of pneumonia following a 1-week course of high dose intravenous Unasyn^®^ (ampicillin sodium/sulbactam sodium) evidenced by negative cultures from the tracheal aspirates and blood cultures, he continued to be clinically septic with multiple spikes of the temperature of above 39.5°C. However, it was found the serum lactate was persistently within the range of 2.0–4.3 mmol/L, suggestive of ongoing sepsis; furthermore, other septic parameters such as procalcitonin and C-reactive protein (CRP) did not improve despite antibiotics (Table 1). The consciousness level also worsened during this episode of sepsis with a fluctuating GCS between 7 and 10. A full septic workup including a cerebral spinal fluid (CSF) culture was taken from the indwelling catheter of the EVD and was found to be positive for a *P. putida*, sensitive to ceftazidime with a minimum inhibitory concentration (MIC) of 1.5.

**TABLE 1:** Range of infective markers during ICU stay.

Parameters	Day 1	Day 7	Day 14	Day 21	Day 28	Day 35	Day 42
White blood cell count (× 10^9^/L)	7.2	3.9	17.3	15.0	21.7	19.1	8.7
C-reactive protein (mg/dl)	0.91	19.27	25.56	28.11	33.78	25.71	2.33
Procalcitonin (ng/ml)	0.14	0.22	0.43	1.55	2.23	1.15	0.23
Lactate (mmol/L)	1.1	2.3	2.7	1.9	4.3	3.1	0.9

Following treatment with intravenous ceftazidime for 1 week and a revision of the EVD on day 32 of admission, his clinical condition improved with a GCS of 14 and was able to be weaned off from mechanical ventilation. A repeated CT brain excluded further complications or any new infections originating from the central nervous system (CNS). Infective markers including white cell count, CRP, and procalcitonin level showed significant reduction (Table 1). He was shifted from ICU care to the neurosurgical ward with supplemental oxygen on day 42 of admission for further rehabilitation.

## Discussion

Ventriculitis often complicates external shunts and catheters involving the CNS which includes the commonly used EVD system in many neurosurgical cases with infection being one of the most common complications of this procedure where infection rates vary between 3.9 and 19% (3). The common pathogens that cause ventriculitis identified are coagulase-negative staphylococcus (62%) followed by *Enterococcus* sp. (19%) (4). Scheithauer et al. (5) reported that coagulase-negative staphylococci were the main pathogen (56%) responsible for meningitis, followed by *Staphylococcus aureus* (25%). A recent larger study done in 2021 states that the main pathogens were streptococci (44.9%), Gram-negative bacilli (27.6%), and staphylococci (15.3%) (6). Gram-negative ventriculitis is associated with a worse outcome in comparison with Gram-positive ventriculitis with mortality rates from 8 to 70% in more severe cases (7).

In our intensive care setting, *P. putida* is found in the environment together with other Gram-negative bacteria which include *Acinetobacter* sp. and *Stenotrophomonas* sp. It can lead to nosocomial infections, especially in patients who are immunocompromised as well as those with invasive catheter placement and medical devices such as EVD (2, 8). Despite clinical data regarding *P. putida* infection being scarce given the low virulence of this organism, it is important to highlight its potential to cause nosocomial infection in our ICU settings and can develop MDR strains to most beta-lactam antibiotics. *P. putida* infection may also occur in immunocompetent patients, thus a high index of suspicion is very important for the diagnosis. Furthermore, CSF biochemistry can be indistinguishable from other forms of meningitis or ventriculitis. Therefore, CSF Gram staining and culturing are crucial in all patients with a suspected diagnosis of CNS infections.

Compared to *Pseudomonas aeruginosa* isolates, *P. putida* isolates were generally considered to have a low level of virulence and to be of little clinical significance. A series of cases of *P. putida* bacteremia was described by Yoshino et al. (8) in 2011 over 4 years with a total of 28 reports. It is typically associated with an indwelling device (61.9%) or immunocompromised state (85.7%). However, the prognosis of *P. putida* bacteremia is excellent following a targeted course of antibiotics with 92.9% cured with appropriate antimicrobial therapy and good source control which is the removal or exchange of the indwelling catheter (2, 8).

*Pseudomonas putida* is recognized as a rare pathogen of meningitis in adult patients and is usually associated with indwelling CNS catheters. The presence of a solid tumor was the most common underlying disease related to the *P. putida* infection (*n* = 8, 44%), followed by traumatic intracranial hemorrhage (*n* = 2, 11%). A total of 44% of patients underwent surgery prior to diagnosis and 11% had an immunocompromised state with 89% of patients having exposure to antibiotics 1 month prior to infection. Most cases were related to indwelling catheters (2). Following this, the strategy that one could apply to prevent *P. putida* infection would be a strict aseptic technique when handling indwelling catheters. Cohort nursing is also important especially in the ICU setting to prevent cross infection between patients. Unnecessary peripheral lines or catheters should be removed promptly as it is a source of infection. If a *P. putida* infection has been isolated, a discussion with a clinical microbiologist for early commencement of appropriate antibiotics would be beneficial to prevent further complications.

## Conclusion

Thus, it is necessary to be aware of the possibility of nosocomial *P. putida* infection, especially in patients with indwelling catheters, and to consider the early initiation of appropriate antibiotic regimens once detected as well as strict precautions in hygiene during the management of these patients to avoid further development of MDR strains.

## Data availability statement

The raw data supporting the conclusions of this article will be made available by the authors, without undue reservation.

## Ethics statement

Ethical review and approval was not required for the study on human participants in accordance with the local legislation and institutional requirements. The patients/participants provided their written informed consent to participate in this study. Written informed consent was obtained from the individual(s) for the publication of any potentially identifiable images or data included in this article.

## Author contributions

MM, IA, and FA contributed in acquisition of data, relevant investigations, getting informed consent from the patient, and helping out in writing up the manuscript. RA and AI edited, critically revised, and proofread the manuscript. All authors contributed to the article and approved the submitted version.
